# Multimodal measurement of glycocalyx degradation during coronary artery bypass grafting

**DOI:** 10.3389/fmed.2022.1045728

**Published:** 2022-11-29

**Authors:** Martine E. Bol, J. B. Huckriede, K. G. H. van de Pas, T. Delhaas, R. Lorusso, G. A. F. Nicolaes, J. E. M. Sels, M. C. G. van de Poll

**Affiliations:** ^1^Department of Intensive Care Medicine, Maastricht University Medical Center (MUMC+), Maastricht, Netherlands; ^2^School of Nutrition and Translational Research in Metabolism (NUTRIM), Maastricht University, Maastricht, Netherlands; ^3^Department of Biochemistry, Cardiovascular Research Institute Maastricht (CARIM), Maastricht University, Maastricht, Netherlands; ^4^Department of Biomedical Engineering, Cardiovascular Research Institute Maastricht (CARIM), Maastricht University, Maastricht, Netherlands; ^5^Department of Cardio-Thoracic Surgery, Maastricht University Medical Center (MUMC+), Maastricht, Netherlands; ^6^Department of Cardiology, Maastricht University Medical Center (MUMC+), Maastricht, Netherlands; ^7^Department of Surgery, Maastricht University Medical Center (MUMC+), Maastricht, Netherlands

**Keywords:** glycocalyx, microcirculation, CABG, cardiopulmonary bypass, endothelial activation

## Abstract

**Background:**

Glycocalyx shedding and subsequent endothelial dysfunction occur in many conditions, such as in sepsis, in critical illness, and during major surgery such as in coronary artery bypass grafting (CABG) where it has been shown to associate with organ dysfunction. Hitherto, there is no consensus about the golden standard in measuring glycocalyx properties in humans. The objective of this study was to compare different indices of glycocalyx shedding and dysfunction. To this end, we studied patients undergoing elective CABG surgery, which is a known cause of glycocalyx shedding.

**Materials and methods:**

Sublingual glycocalyx thickness was measured in 23 patients by: 1) determining the perfused boundary region (PBR)—an inverse measure of glycocalyx thickness—by means of sidestream dark field imaging technique. This is stated double, 2) measuring plasma levels of the glycocalyx shedding products syndecan-1, hyaluronan, and heparan sulfate and 3) measuring plasma markers of impaired glycocalyx function and endothelial activation (Ang-2, Tie-2, E-selectin, and thrombomodulin). Measurements were performed directly after induction, directly after onset of cardiopulmonary bypass (CPB), and directly after cessation of CPB. We assessed changes over time as well as correlations between the various markers.

**Results:**

The PBR increased from 1.81 ± 0.21 μm after induction of anesthesia to 2.27 ± 0.25 μm (*p* < 0.0001) directly after CPB was initiated and did not change further during CPB. A similar pattern was seen for syndecan-1, hyaluronan, heparan sulfate, Ang-2, Tie-2, and thrombomodulin. E-selectin levels also increased between induction and the start of CPB and increased further during CPB. The PBR correlated moderately with heparan sulfate, E-selectin, and thrombomodulin and weakly with Syndecan-1, hyaluronan, and Tie-2. Shedding markers syndecan-1 and hyaluronan correlated with all functional markers. Shedding marker heparan sulfate only correlated with Tie-2, thrombomodulin, and E-selectin. Thrombomodulin correlated with all shedding markers.

**Conclusion:**

Our results show that glycocalyx thinning, illustrated by increased sublingual PBR and increased levels of shedding markers, is paralleled with impaired glycocalyx function and increased endothelial activation in CABG surgery with CPB. As correlations between different markers were limited, no single marker could be identified to represent the glycocalyx in its full complexity.

## Introduction

The microcirculation is compromised during numerous disease states, such as sepsis, shock, critical illness, and major surgery (1–5). Microcirculatory dysfunction may include increased flow heterogeneity and decreased vascular density (6). A known cause of microcirculatory dysfunction is coronary artery bypass graft (CABG) surgery in which microcirculatory dysfunction has been linked to postoperative increased lactate levels and elevated acute organ injury scores (7–11). In patients undergoing major abdominal surgery, as well as in septic patients and shock patients, microcirculatory derangements have been associated with impaired organ function and impaired outcomes (3, 5, 12–14). As such, protection and restoration of the microcirculation shows great promise to reduce complications and improve outcomes (15).

A key component of the microcirculation that is affected is the glycocalyx (16, 17). The glycocalyx is a gel-like layer lining the luminal side of vascular endothelial cells. It is a network of proteoglycans, glycosaminoglycans, and glycoproteins with non-covalently linked endothelium- and plasma-derived molecules therein (18, 19). The glycocalyx forms a primary barrier between blood constituents and vascular endothelium, thereby preventing leakage of plasma components, inhibiting platelet activation, regulating signaling molecules, and regulating leukocyte adhesion (18, 20).

Despite the importance of the glycocalyx in (patho)physiology and its potential as a target in the prevention of end-organ damage, there is no golden standard for its assessment (21). Generally, one of the following methods is used for glycocalyx measurements: (1) measurement of the sublingual glycocalyx dimensions using a video microscope, (2) measurement of degradation markers (glycocalyx constituents) in plasma, and (3) measurement of functional aspects of the glycocalyx/endothelial activation such as markers of vessel wall permeability. Different studies, among which some were performed during CABG surgery, reported different subsets of variables (16, 17, 22–24). This complicates comparisons between studies and the establishment of associations between different markers.

In this study, we evaluated glycocalyx dynamics through all three measurement methods simultaneously to gain more insights into glycocalyx (patho)physiology and how the different measurement methods relate to each other. We studied patients undergoing CABG surgery with extracorporeal circulation and induced cardiac ischemia as they form a predictable, *in vivo* model of endothelial glycocalyx dysfunction.

## Materials and methods

A representative sample of adult patients (≥18 years) that undergo elective CABG surgery with the use of cardiopulmonary bypass (CPB) were considered eligible for inclusion. Patients were included if an observer and equipment were available for performing measurements. The only exclusion criteria that were applied were related to the technical feasibility of sublingual SDF imaging (i.e., no oral injuries or infections). The study protocol was approved by the medical ethics committee of the Maastricht University Medical Center (MUMC+) and written informed consent was obtained from all participants before inclusion. To obtain a complete dataset during CABG surgery, patients were excluded from the study in case not all measurements could be performed.

### Measurements

Anesthesia was induced and maintained with propofol, midazolam, sufentanil, and rocuronium, after which the sternum was opened and vessels (generally the left internal thoracic artery and the great saphenous vein) were prepared for coronary grafting. Cardiac arrest was established after aortic cross-clamping by administration of a potassium-rich cardioplegia solution either added to a crystalloid solution (St. Thomas cardioplegia) or blood (blood cardioplegia) according to the surgeon’s preference. A heart-lung machine with a roller pump and a heater-cooler device (Sorin Stockert S5, Livanova, Mirandola, Italy) was used to conduct CPB with heparin-coated circuits. Heparin was administered prior to the start of CPB to achieve an activated clotting time (ACT) of at least 400 s. After cessation of the CPB, the effect of heparin was reversed with protamine sulfate (1 mg of protamine for every 100 units of heparin). Shed blood, collected during both the heparinized and non-heparinized phase of surgery and buffered in a cell saver reservoir, was transfused into the patient at the end of surgery. Additionally, cardiotomy suction was performed. Normothermia was maintained during surgery. After surgery, patients were admitted to the intensive care unit (ICU).

Data were collected at four points in time: (1) directly after induction of anesthesia (baseline), (2) directly after start of CPB, (3) directly after cessation of CPB, and (4) 2 h after surgery at the ICU. Study measurements did not alter standard surgical procedures as performed in our center.

#### Sublingual glycocalyx measurements

At all four time points, multiple sublingual microcirculation movies of 1 s (23 frames) were acquired with an SDF camera (CapiScope HVCS, KK Technology, Honiton, UK) fitted with GlycoCheck software (Microvascular Health Solutions Inc., Salt Lake City, UT, USA) to obtain a single measurement for each time point. At each time point, three consecutive measurements were performed with the GlycoCheck as previous research by us and others indicated this to be crucial to achieve reliable results (25, 26).

**FIGURE 1:**
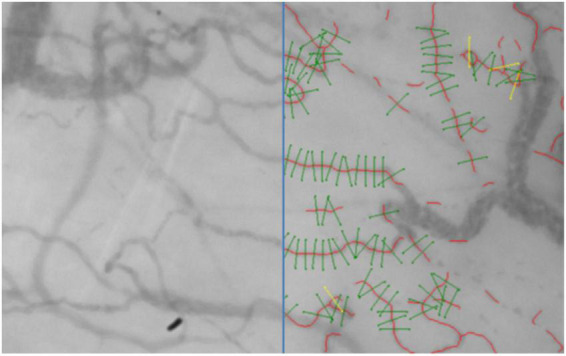
A frame recorded with an SDF camera with on the right an overlay of the vessels (red) with valid measurement points (green) that were detected and analyzed by the GlycoCheck software.

**FIGURE 2:**
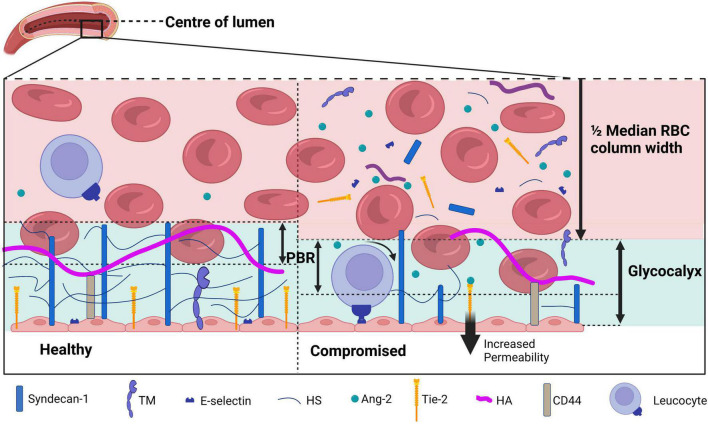
A schematic overview of the glycocalyx under physiological **(left)** and diseased **(right)** conditions covering the endothelial cells in the lumen of a blood vessel. The perfused boundary region (PBR) is defined as the distance between the median red blood cell (RBC) column width and the limit where erythrocytes can be found. During physiological conditions, the glycocalyx is composed of syndecan-1 and glycosaminoglycans including heparan sulfate (HS) and hyaluronan (HA). Receptors such as thrombomodulin (TM), Tie-2, and E-selectin are present on the endothelial membrane. During disease, the glycocalyx is damaged and components are shed to the plasma, which causes an increase in the PBR as erythrocytes are able to penetrate deeper in the glycocalyx layer. Activation of endothelial cells causes both activation and release of surface receptors TM, Tie-2, and E-selectin. E-selectin activation increases leukocyte rolling, increased Ang-2 levels binding to Tie-2 increases vessel permeability. Figure created with www.biorender.com.

Analysis of the sublingual movies per time point was performed with GlycoCheck software. GlycoCheck’s measurement method is described by Lee et al. (27). In brief, measurement points are defined at 10 μm intervals on automatically detected vessels with a diameter between 5 and 25 μm (Figure 1). A single measurement was complete when at least 3,000 measurement points had been acquired. Sublingual glycocalyx thickness is calculated based on the assumption that the glycocalyx can roughly be divided into two sublayers: an inner layer that is penetrable to red blood cells—the so-called perfused boundary region (PBR)—and an outer layer that is impenetrable to red blood cells. As an intact glycocalyx is less penetrable to red blood cells compared to a damaged glycocalyx, the PBR can be used as an inverse measure of glycocalyx thickness (Figure 2). The measurement time was limited to 15 min at each time point; if measurements were not completed at that time, they were registered as missing data. Reasons for prolonged sublingual measurements include large amounts of saliva or debris in the mouth and excess lingual movement.

#### Glycocalyx markers

Directly after induction of anesthesia, directly after onset of CPB and directly after CPB was ceased, blood samples were collected from a radial, arterial line in citrate tubes and centrifuged twice at 4,000 RPM for 12 min at room temperature. Plasma was stored at −80°C and later analyzed to determine glycocalyx shedding products and markers that indicate glycocalyx function and endothelial activation. To determine glycocalyx shedding products, commercial enzyme-linked immunosorbent assays (ELISAs) were used according to the manufacturer’s instructions for Syndecan-1 (DuoSet ELISA, R&D systems, Bio-Techne, Minneapolis, USA), hyaluronan (DuoSet ELISA, R&D systems, Bio-Techne, Minneapolis, USA) and heparan sulfate (Elabscience Biotechnology Inc., Houston, Texas, USA). To determine glycocalyx function and endothelial activation, Angiopoietin-2 [a marker that reflects vascular permeability (28)], Tie-2 [a marker that reflects vascular instability, including endothelial barrier function and inflammation (29)], E-selectin [a marker expressed on activated endothelium to mediate rolling leukocyte adhesion (30)], and thrombomodulin [an anticoagulant mediator expressed by endothelial cells that can bind to the glycocalyx (31)] were measured with commercial ELISAs from R&D systems (DuoSet ELISA, Bio-Techne, Minneapolis, USA). Plasma samples were diluted 1:10 for R&D systems and 1:100 for Elabscience in 1% BSA reagent diluent. All plasma measurements were performed in triplicate, averaged, and corrected for plasma dilution during surgery with respect to the levels measured directly after induction by correcting for hemoglobin levels.

### Statistical analysis

Data analysis was performed using GraphPad Prism (Version 9.2.0; GraphPad Software, San Diego, CA, USA). Normality was assessed using the Kolmogorov-Smirnov test for normality. Data are presented as mean ± standard deviation (SD) for normally distributed data and as median [25th–75th percentile] for non-normally distributed data. Outliers were identified based on the Tukey method. Differences between time points were tested using the repeated measures ANOVA or the Friedman test as appropriate. *Post-hoc* testing was performed using a paired t-test or Wilcoxon signed-rank test with Bonferroni correction. Correlations between variables were calculated using Spearman rank correlation tests. A Spearman’s rho below 0.2 was considered negligible, between 0.2 and 0.4 was considered weak, between 0.4 and 0.6 was considered moderate, between 0.6 and 0.8 was considered strong and above 0.8 was considered very strong. *P*-values < 0.05 were considered statistically significant.

## Results

### Study population

Of the 35 patients measured, 12 were excluded from the study for not having a full set of measurements. Baseline characteristics of the 23 included patients are shown in Table 1. The included population shows classical characteristics for patients that undergo elective CABG surgery, meaning 35% of patients were diabetic, 87% had hypertension and the mean BMI was 28.2 ± 4.9 (32). Hemoglobin levels dropped from 8.0 ± 0.9 mmol/L directly after induction to 5.3 ± 1.1 mmol/L at the start of the CPB and 5.3 ± 0.8 mmol/L when CPB was stopped.

**TABLE 1:** patient characteristics of the 23 patients.

Characteristic	Value
Age (years)	68.0 ± 6.4
Female; *N* (%)	3 (13%)
Height (cm)	172.4 ± 7.6
Weight (kg)	84 ± 15
BMI (kg/m^2^)	28.2 ± 4.9
Hypertension; *N* (%)	20 (87%)
Diabetes; *N* (%)	8 (35%)
Aortic clamp time (minutes)	52 ± 19
Number of grafts	3 (2–4)
Length of ICU stay (days)	2 (2–3)
Length of hospital stay (days)	8 (7–9)
SOFA score the day after surgery	4.4 ± 2.4

BMI, Body Mass Index; ICU, Intensive Care Unit, SOFA, Sequential Organ Failure Assessment.

### Sublingual SDF video camera measurements

**FIGURE 3:**
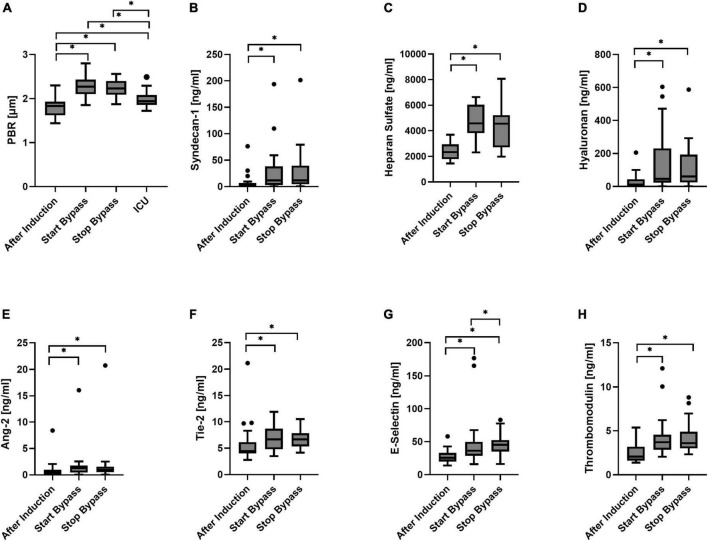
Boxplot of the perfused boundary region [PBR; **(A)**], Syndecan-1 **(B)**, heparan sulfate **(C)**, hyaluronan **(D)**, Angiopoietin-2 [Ang-2, **(E)**], Tie-2 **(F)**, E-selectin **(G)**, and thrombomodulin **(H)** for all time points. *N* = 23 during surgery; *N* = 19 at ICU. *p < 0.05.

The PBR increased from 1.81 ± 0.21 μm at baseline to 2.27 ± 0.25 μm directly after CPB was started (*p* < 0.0001). There was no significant change in PBR between the beginning and the end of CPB (PBR = 2.23 ± 0.19 μm). Two hours after arrival at the ICU the PBR was, compared to its value directly after onset of CPB, significantly decreased to 1.98 ± 0.19 μm, but still statistically significantly higher than the PBR at baseline (Figure 3A).

### Glycocalyx shedding markers

Syndecan-1 was, compared to its value at baseline, statistically significantly increased directly after CPB was started. Syndecan-1 levels at the end of CPB did not differ significantly from the levels measured at the start of extracorporeal circulation (Figure 3B). One patient had an increased level of syndecan-1 at baseline that increased even further during surgery (top outlier of Figure 3B). At the start of CPB, heparan sulfate had increased from baseline. No statistically significant change in heparan sulfate concentrations was observed between the start and end of CPB (Figure 3C). Directly after CPB was started, hyaluronan plasma concentrations increased significantly with respect to baseline. No significant change was observed during CPB (Figure 3D).

### Markers indicating glycocalyx function and endothelial activation

At the start of CPB, Ang-2 concentrations were increased from baseline. No significant change in Ang-2 concentrations was observed between the start and end of CPB (Figure 3E). A single patient showed high levels of Ang-2 at baseline that increased further during surgery (outliers of Figure 3E); this was not the same patient that showed relatively high syndecan-1 levels. At the onset of CPB, Tie-2 concentrations were significantly increased with respect to baseline. Concentrations of Tie-2 at the start and end of CPB did not differ significantly (Figure 3F). Directly after onset of CPB, E-selectin concentrations were increased significantly from baseline. During CPB the E-selectin concentrations significantly increased further (Figure 3G). Directly after CPB was started, the concentrations of thrombomodulin were increased was started with respect to baseline (Figure 3H). There was no statistically significant change between the start and end of CPB.

### Correlations within measurement methods

Table 2 shows the Spearman rank correlation coefficients within and between the different measurement methods. Shedding marker hyaluronan correlated strongly with syndecan-1 and only weakly with heparan sulfate. Syndecan-1 and heparan sulfate did not correlate significantly with each other. Functional markers Ang-2 correlated moderately with Tie-2 and weakly with E-selectin. Thrombomodulin correlated moderately with E-selectin and weakly with Tie-2.

**TABLE 2:** Spearman rho correlation coefficients (top number) and their respective *p*-values (bottom number) for the correlations between measurements.

		SDF camera	Shedding markers			Functional/endothelial activation markers				
		PBR	Syndecan-1	Hyaluronan	Heparan Sulfate	Ang-2	Tie-2	E-selectin	Thrombo-modulin	
SDF camera	PBR		0.38 0.0012	0.29 0.016	0.42 3.9 × 10^–4^	0.11 0.37; NS	0.35 0.0036	0.44 1.4 × 10^–4^	0.41 4.7 × 10^–4^	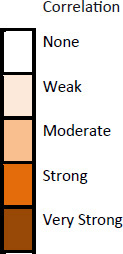
Shedding markers	Syndecan-1	0.38 0.0012		0.60 6.13 × 10^–8^	0.19 0.11; NS	0.35 0.0034	0.36 0.0023	0.40 0.00058	0.66 7.69 × 10^–10^	
	Hyaluronan	0.29 0.016	0.60 6.13 × 10^–8^		0.30 0.013	0.28 0.019	0.28 0.020	0.27 0.0024	0.48 2.61 × 10^–5^	
	Heparan Sulfate	0.42 3.9 × 10^–4^	0.19 0.11; NS	0.30 0.013		0.085 0.49; NS	0.34 0.0043	0.28 0.0020	0.48 3.28 × 10^–5^	
Functional/endothelial activation markers	Ang-2	0.11 0.37; NS	0.35 0.0034	0.28 0.019	0.085 0.49; NS		0.53 2.9 × 10^–6^	0.37 0.0017	0.23 0.054; NS	
	Tie-2	0.35 0.0036	0.36 0.0023	0.28 0.020	0.34 0.0043	0.53 2.9 × 10^–6^		0.52 4.12 × 10^–6^	0.29 0.015	
	E-selectin	0.44 1.4 × 10^–4^	0.40 0.00058	0.27 0.0024	0.28 0.0020	0.37 0.0017	0.52 4.12 × 10^–6^		0.48 2.45 × 10^–5^	
	Thrombo-modulin	0.41 4.7 × 10^–4^	0.66 7.69 × 10^–10^	0.48 2.61 × 10^–5^	0.48 3.28 × 10^–5^	0.23 0.054; NS	0.29 0.015	0.48 2.45 × 10^–5^		

### Correlation between measurement methods

The PBR correlated moderately with the shedding marker heparan sulfate and the functional markers E-selection and Thrombomodulin. A weak correlation was observed between PBR and syndecan-1, hyaluronan, and Tie-2. Thrombomodulin correlated strongly with shedding marker Syndecan-1 and moderately with the other shedding markers hyaluronan and heparan sulfate. Shedding marker syndecan-1 was moderately correlated with functional marker E-selectin. Other correlations between markers of glycocalyx dysfunction and glycocalyx shedding were weak or absent (Ang-2 vs. heparan sulfate) (Table 2). Thrombomodulin and E-selectin were the only markers that correlated with any marker of a different aspect of glycocalyx injury.

## Discussion

In this paper we investigated glycocalyx damage during CABG surgery with CPB through three measurement methods simultaneously: indirect visualization using a video microscope, glycocalyx shedding product concentrations, and markers indicating glycocalyx function and endothelial activation. The aim of our study was to investigate how these different measurements relate to each other during CABG surgery as this is a well-known cause of glycocalyx damage. A pattern of early damage was present directly after the onset of CPB and neither further damage nor recovery was observed for all three measurement methods. Only the systemic inflammation marker E-selectin (33, 34), an adhesion receptor that mediates leukocyte rolling on the endothelium showed an additional increase during CPB.

Our study shows that an increase in PBR and glycocalyx shedding product levels was paralleled with impaired glycocalyx/endothelial function and increased endothelial activation. This suggests a close relationship between glycocalyx thickness and vascular endothelial function during CABG surgery with CPB. This is in line with the perception that the endothelial glycocalyx serves as a key factor in maintaining homeostasis of the vascular endothelium (35, 36). Even though a causal relationship cannot be established from our data, some evidence suggests that glycocalyx damage at least precedes impaired endothelial function as Richter et al. showed that in septic children and in mouse models of sepsis, heparan sulfate levels peak prior to Ang-2 levels (37). Elevated Ang-2 concentrations have been associated with increased albuminuria which is associated with impaired pulmonary and renal function (38, 39). Additionally, endothelial dysfunction as reflected by increased thrombomodulin concentrations was associated with increased duration and severity of acute kidney injury following severe trauma (40). As such, preservation of glycocalyx integrity may reduce peri- and post-operative organ dysfunction and complications.

Our data further shows glycocalyx damage, as reflected by all three measurement methods, to be present directly after the onset of CPB and to persist, but not worsen, during CPB. Potential sources of glycocalyx damage between post-induction of anesthesia and the first minutes of CPB flow include surgical trauma, large amount of fluid administration (including CPB priming fluid), and initiation of CPB (including contact blood activation) (9). The timeframe of our measurements does not allow us to identify which of these aspects contribute to the observed glycocalyx degradation and dysfunction. Our study aim, however, was not to determine factors that damage the glycocalyx, but to compare different measurement methods. For this aim we needed glycocalyx shedding to take place between our measurement points, which it did according to our data. As such the chosen time points were in line with our aim.

A strength of our study is the fact that we performed simultaneous measurements allowing for a comparison between the different outcome measures (9). As surgical techniques and anesthesiologic procedures vary between medical centers, it is not evident that markers can be compared between different studies. Factors, such as whether pulsatile CPB flow was used or not, have been shown to potentially influence glycocalyx dynamics (7, 16).

Most measurements correlated only weakly with each other implying no single measurement could suffice to comprehensively describe glycocalyx dynamics. Thrombomodulin, a marker that indicates impaired glycocalyx/endothelial activation, showed the best overall correlations with all shedding markers and the PBR. This implies that it should be chosen if only a single measurement is to be used. Classically, thrombomodulin is considered to be a cofactor for thrombin binding mediating protein C activation and inhibiting thrombin activity and hence, thereby functioning as an anticoagulant (41). In recent years, thrombomodulin is increasingly acknowledged to exhibit more properties such as an anti-inflammatory function (42, 43). Recent studies have shown thrombomodulin levels to be of specific interest as it proves to be a valuable prognostic marker associated with increased mortality rate and disease progression (44).

Syndecan-1, the most widely reported marker representing glycocalyx integrity (45), proved to be the shedding marker showing the highest correlations with the functional aspects of endothelial glycocalyx. PBR correlated moderately at most with all three shedding markers and with all functional markers but Ang-2. This implies that both syndecan-1 and thrombomodulin levels result in a better overview of glycocalyx integrity. PBR, however, is a non-invasive point-of-care measurement that can provide bed-side glycocalyx monitoring. Due to its ease of use and rapid results it may still be regarded as a valuable glycocalyx measurement method.

A limitation of our study is that we did not also perform measurements before anesthesia. However, Dekker et al. showed that PBR, syndecan-1, and heparan sulfate did not differ before and after induction of anesthesia (16) and additionally our measured PBR and marker concentrations reflect levels consistent with no or very limited elevation (46). Another limitation of our study is the fact that we compared circulating plasma markers with the PBR measured sublingually with an SDF camera. It cannot be guaranteed that measurements performed sublingually correctly reflect systemic changes in glycocalyx integrity although evidence suggests that in case of systemic changes in glycocalyx dimensions, the sublingual glycocalyx dimensions change accordingly (47–49). As the sublingual region is easily accessible, providing a non-invasive measurement, it is widely used to study microcirculatory parameters such as the glycocalyx (50, 51). The PBR is not based on direct visualization of the glycocalyx thickness, but based on the assumption that impaired glycocalyx is more permeable when damaged (52). Despite the mentioned limitations of the PBR, its potential value has been underlined by studies that link increased PBR with increased disease severity and worse clinical outcomes such as organ damage and mortality in different populations (12, 53).

To our knowledge, we are the first to simultaneously evaluate glycocalyx integrity through sublingual video microscopy, different shedding markers, and multiple functional markers. Our results show glycocalyx thinning to be paralleled with impaired glycocalyx function and increased endothelial activation in CABG surgery with CPB. As correlations between different markers were limited, no single marker could be identified to represent the glycocalyx in its full complexity. It is important to realize in future research that different markers represent different aspects of glycocalyx injury and are to be used complementary.

## Data availability statement

The raw data supporting the conclusions of this article will be made available by the authors upon request, without undue reservation.

## Ethics statement

The studies involving human participants were reviewed and approved by the Medical Ethics Committee of the Maastricht University Medical Center (MUMC+). The patients/participants provided their written informed consent to participate in this study.

## Author contributions

MB and JH performed the data acquisition and analysis. KP performed data acquisition. All authors contributed to designing the study, drafting the manuscript, and substantially revising the manuscript.
